# The Safety of Consuming Water Dropwort Used to Purify Livestock Wastewater Considering Accumulated Antibiotics and Antibiotic Resistance Genes

**DOI:** 10.3390/antibiotics11040428

**Published:** 2022-03-23

**Authors:** Dongrui Yao, Yajun Chang, Wei Wang, Linhe Sun, Jixiang Liu, Huijun Zhao, Weiguo Zhang

**Affiliations:** 1 Jiangsu Key Laboratory for the Research and Utilization of Plant Resources, Institute of Botany, Jiangsu Province and Chinese Academy of Sciences (Nanjing Botanical Garden Memorial Sun Yat-Sen), Nanjing 210014, China; yaodongrui@cnbg.net (D.Y.); changyj@cnbg.net (Y.C.); izjwang@163.com (W.W.); linhesun@cnbg.net (L.S.); ljx891654338@163.com (J.L.); 2 College of Geography and Environmental Science, Northwest Normal University, Lanzhou 730070, China; zhaohj0707@163.com; 3 Institute of Agricultural Resources and Environment, Jiangsu Academy of Agricultural Sciences, Nanjing 210014, China

**Keywords:** antibiotic, antibiotic resistance gene, health risk, water dropwort, livestock wastewater

## Abstract

Research is lacking on the health risks of antibiotics and antibiotic resistance genes (ARGs) in water dropwort grown in livestock wastewater. Our results showed that antibiotics from livestock wastewater were absorbed and bioaccumulated by water dropwort. The concentration of antibiotics was higher in the roots than in the stems and leaves. The health-risk coefficients of antibiotics in water dropwort were below the threshold (<0.1), indicating that in this case study, the consumption of water dropwort used to purify livestock wastewater was safe for humans considering accumulated antibiotics. ARGs were closely correlated between livestock wastewater and water dropwort, with the results showing that all 13 ARGs detected in the livestock wastewater were also found in the water dropwort. Tetracycline resistance genes were more abundant than the other ARGs in both the livestock wastewater and water dropwort. The estimated daily intake of ARGs in water dropwort for humans ranged from 2.06 × 10^6^ to 7.75 × 10^12^ copies g^−1^_,_ suggesting the potential risk of intaking ARGs in water dropwort cannot be ignored. Although the safety of consuming water dropwort used to purify livestock wastewater, considering accumulated antibiotics and ARGs, was assessed in this study, more studies should be conducted to ensure we fully understand the health risks.

## 1. Introduction

As the world’s most populous country, China has a huge demand for high-quality meat, eggs, and milk [1]. Large-scale livestock and poultry farming has rapidly developed, resulting in the production of livestock waste of up to 200 million tons per year, which has massive potential to cause pollution [2,3]. Therefore, phytoremediation has been widely applied to livestock wastewater [4,5]. The misuse of antibiotics in livestock breeding has caused antibiotic pollution in livestock waste [6,7]. Antibiotic residues in the environment can bioaccumulate in vegetables and crops, posing a threat to human health [8,9,10,11]. Moreover, an environment contaminated by antibiotics can exert selective pressure and lead to the emergence and enrichment of antibiotic resistance genes (ARGs). ARGs can be disseminated among microorganisms through vertical and horizontal gene transfer [12,13]. One Health emphasizes the organic unity of human, animal, and environmental health. Antibiotics and ARGs can accumulate and disseminate in humans, animals, and the environment, posing a risk to One Health. A growing number of studies have detected ARGs in a variety of agricultural products [14,15,16,17]. The application of livestock waste can increase ARGs in vegetables, indicating that ARGs migrate from contaminated environments to vegetables. Once the vegetables contaminated with ARGs are ingested by consumers, clinical pathogens of human or animals might acquire resistance to antibiotics, making some diseases more difficult to cure.

Water dropwort (*Oenanthe javanica* (Bl.) DC.) is a plant that can efficiently remove nitrogen and phosphorus from livestock wastewater as well as tolerate freezing temperatures [18,19]. Moreover, water dropwort is an edible vegetable that is a favorite in southeast Asia and China [20,21]. Phytoremediation of livestock wastewater with water dropwort can provide considerable ecological and economic benefits. Therefore, water dropwort has often been planted to purify and recycle livestock wastewater in recent years [22]. However, when plants absorb nitrogen and phosphorus from livestock wastewater, antibiotics are gradually accumulated. The antibiotics accumulated in plants induce a selective pressure of ARGs on the endophytic bacteria, which can also acquire exogenous ARGs from the growth environment. If these plants are edible vegetables, they can pose a potential risk to consumer health. Therefore, it is urgent to assess the safety of consuming water dropwort used to purify livestock wastewater considering accumulated antibiotics and antibiotic resistance genes. This was the main objective of this study.

## 2. Materials and Methods

### 2.1. Experimental Design and Samples Collection

Livestock wastewater was collected from a cattle farm in Suqian City, Jiangsu Province, China (33°120′~34°250′ N, 117°60′~119°130′ E). The cattle farm where 1052 cows were fed covered 20.7 acres. Cattles Three’s cows were in a barn (5 × 4 m). The cows were fed mainly foraged grass supplemented by commercial feed. After flushing, cow manure was transferred to an ant-seepage pool (80 × 80 × 2 m). After wet and dry separation, the liquid part (wastewater) was placed into cement pools (10 × 5 × 1.6 m), where water dropwort was planted in floating beds (6 × 6 m) on 16 May 2021 and harvested on 25 July 2021. The sterilized nutrient solution was used to grow water dropwort seedlings. Before transplanting, the antibiotics and ARGs in water dropwort seedlings were detected to rule out the effects of self-carrying antibiotics and ARGs. The determination method is described in Section 2.2. Only water dropwort seedlings without these contaminants were used in this study. There was a total of 20 pools at the farm. Seventeen pools, with a floating bed in each pool, were used to grow water dropwort. The control group was the remaining 3 pools where no water dropwort was planted, and their role in this research was to provide the water samples. Three of the seventeen pools were randomly selected to collect water dropwort samples. Twenty water dropwort plants with similar growth characteristics were randomly collected from each pool. The size of each sample was determined by a ruler and a vernier caliper in order to screen for water dropwort plants with similar growth. Meanwhile, livestock water samples were collected from the pools without water dropwort growth (0–30 cm depth). In detail, three sites in each pool were set to collect livestock wastewater. The distance between the sites was at least 2 m. The livestock wastewater samples from the same pool were mixed together. After being placed into an icebox, the water and plant samples were transported to the laboratory. All the samples were immediately pretreated to prepare for the determination of antibiotics and DNA extraction. In general, the group of wastewater contained the water samples collected from 3 pools with 3 sampling sites each; the group of water dropwort (stem and root) contained the plants samples collected from 3 pools with the collection of 20 water dropwort plants each.

### 2.2. Determination Methods of Antibiotics

Water samples were simultaneously concentrated using solid-phase extraction (SPE, Oasis HLB cartridges, Waters, MA, USA) coupled with ultra-high-performance liquid chromatography tandem mass spectrometry (UHPLC-MS/MS). Detailed information about the procedures, including UHPLC-MS/MS parameters, was presented in a previously published method [23]. Plant tissue samples were freeze-dried and ground to fine particles, 0.5 g of which was used for the analysis of antibiotics. Antibiotics in samples were extracted first by liquid-solid extraction using acetonitrile and then by solid-phase extraction using Oasis HLB extraction cartridges (Waters, Milford, MA, USA), according to Yang et al. [24]. The extract was passed through a 0.22 µm membrane filter, and the concentration of antibiotics was analyzed using an ultra-performance liquid chromatography tandem mass spectrometer, according to Gros et al. [25].

### 2.3. DNA Extraction and Real-Time Fluorescent Quantitative Polymerase Chain Reaction (RT-qPCR)

DNA was extracted from 100 mL livestock wastewater samples using a FastDNA SPIN Kit (MP Bio, Santa Ana, CA, USA). Three water dropwort plants from the same pool were placed into a 1000 mL beaker containing 700 mL sterilized phosphate-buffered saline (0.01 M, pH 7.4) and sonicated for 10 min. Flasks were shaken for 0.5 h at 150 rpm and 30 ℃. After being washed, the roots were separated from stems and leaves with a sterilized blade. The methods for DNA extraction from roots, stems, and leaves followed those described by Mei et al. [14]. RT-qPCR was used to determine the abundance of ARGs. The detailed information of the 13 primer sets is provided in Table 1. PCR amplification was conducted using an ABI-7500 (Applied Biosystems, Inc., Carlsbad, CA, USA). The parameters of amplification and the analysis of results followed the protocol used by Su et al. [26]. The absolute abundance of ARGs was normalized to the number of 16S rRNA gene copies [27,28].

### 2.4. Potential Health Risk Assessment

The bioaccumulation factor (BAF) was used to quantify the antibiotic enrichment capacity of the roots and stems/leaves of the water dropwort compared to the livestock wastewater. The detailed calculation was described by Mei et al. [14].

BAF = C_plant_/C_wastewater_ (1)

where C_plant_ is the concentration of roots or stems/leaves and C_wastewater_ is the concentration of livestock wastewater.

The ratios of estimated daily intake (EDI) to acceptable daily intake (ADI) for each antibiotic were calculated to assess their respective potential risks to adults and children. At present, the hazard quotient (the ratio of EDI to ADI) is still used to evaluate the risk of antibiotics, and the value ≥ 0.1 was set as posing a threat to human health [29,30,31,32]. The detailed calculations of the potential health risks of antibiotics were described by Hanna et al. [32]. The estimated daily intake of ARGs (EI_ARGs_) when consuming water dropwort was calculated following Mei et al. [14].

EI_ARGs_ = GCN/g × W_intake_ (2)

where GCN/g is the gene copy number of ARGs per gram of water dropwort and W_intake_ is the weight of vegetable daily intake.

### 2.5. Statistical Analysis

Data were analyzed with the statistical software SPSS 26.0 (SPSS Inc., Chicago, IL, USA). The mean comparison was performed between wastewater, roots, and stems and leaves. To find the significant differences between wastewater, roots, and stems and leaves and to eliminate type I and type II error, a one-way analysis of variance (ANOVA) with Duncan’s test was used (*p* < 0.05). The heatmap was visualized in the R software environment with the pheatmap package [33]. The other figures were constructed in Origin 9.0 (OriginLab Corporation, Northampton, MA, USA). 

## 3. Results and discussion

### 3.1. Bioaccumulation of Antibiotics in Water Dropwort and Potential Health Risks to Humans

**Figure 1:**
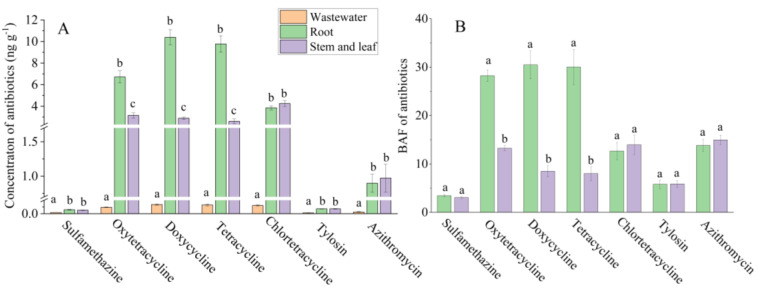
The concentrations of antibiotics in livestock wastewater and water dropwort (**A**). The antibiotic enrichment capacities of roots and stems and leaves (**B**). Different letters indicate significant differences between livestock wastewater, roots, and stems and leaves (*p* < 0.05). BAF = C_plant_/C_wastewater_, where C_plant_ is the concentration of roots or stems and leaves and C_wastewater_ is the concentration of livestock wastewater.

**Figure 2:**
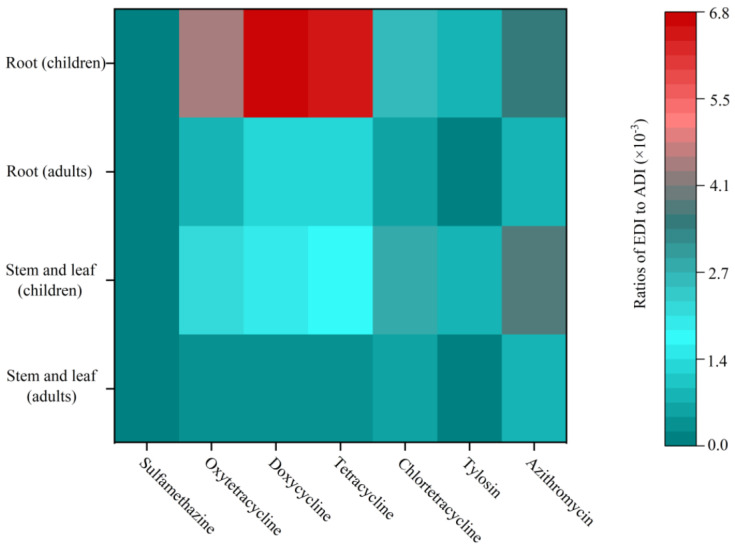
The ratios of estimated daily intake (EDI) to acceptable daily intake (ADI) for each antibiotic. A hazard quotient of ≥0.1 indicates it poses a threat to human health.

China is the world’s largest producer and consumer of antibiotics, 46.1% of which are used in the animal feed industry [11]. Most of these antibiotics are discharged into the environment through livestock waste. Vegetables can absorb and bioaccumulate antibiotics from the environment, which threatens human health if consumed [8,9]. Most consumers in China like to eat the stems and leaves of water dropwort; however, a considerable number of consumers like to eat the roots, which are regarded as a traditional Chinese medicine to cure some chronic diseases such as hypertension, hyperlipidemia, insomnia, etc. [34,35]. Our results showed that both the roots and stems and leaves absorbed and accumulated the antibiotics from the livestock wastewater. The concentrations of tetracyclic antibiotics including oxytetracycline, doxycycline, tetracyclic, and chlortetracycline in roots and stems/leaves were 6.72 and 3.15, 10.39 and 2.89, 9.78 and 2.60, and 3.86 and 4.25 ng g^−1^, respectively. These concentrations were much higher than those of the other antibiotics. (Figure 1A, Table S1). The BAF was used to quantify the antibiotic enrichment capacity of the roots and stems and leaves of the water dropwort compared to that of the livestock wastewater. The BAF analysis showed that the antibiotic enrichment capacity of the roots was much stronger than that of the stems and leaves (Figure 1B, Table S2). Our results verified that the accumulation of antibiotics varied among plant organs [10]. Using the measured accumulation values, the potential health risk of water dropwort to human health was assessed. In this case study, the ratio of EDI to ADI was below the threshold (<0.1), indicating that consuming both the roots and the stems and leaves posed no antibiotic risk to human health (Figure 2). However, further case studies should be conducted to comprehensively evaluate the health risks of water dropwort used to purify livestock wastewater.

### 3.2. ARGs in Water Dropwort and Potential Risk to Human Health

**Figure 3:**
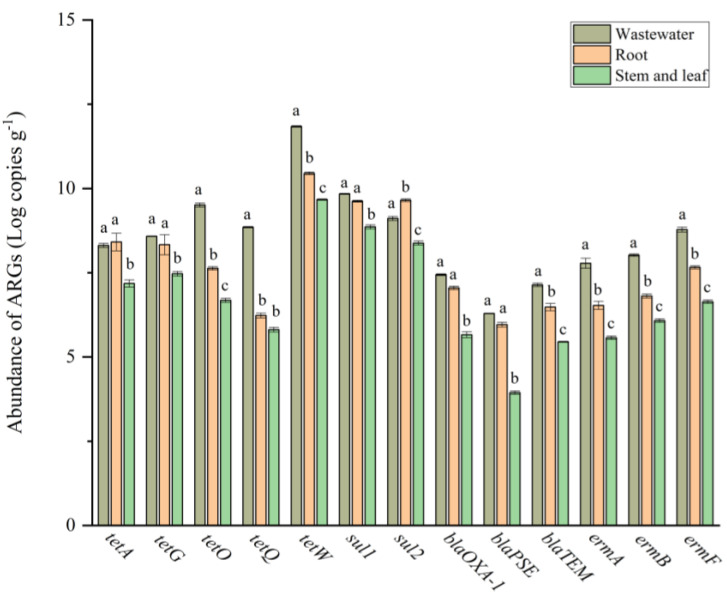
The abundance of antibiotic resistance genes (ARGs) in livestock wastewater and water dropwort. Different letters indicate significant differences between livestock wastewater, roots, and stems and leaves (*p* < 0.05).

A growing number of studies have detected ARGs in a variety of agricultural products [14,15,16,17]. The application of livestock waste can increase the ARGs in vegetables, indicating that ARGs migrate from contaminated environments to vegetables [14,36,37]. This migration mechanism might function via (1) environmental bacteria that colonize roots, migrating up and colonizing the interiors of stems, leaves, and fruits [38]; or (2) the antibiotic-resistant bacteria from water, soil, and air directly attaching to the exterior of vegetables and then colonizing [15,39,40]. Compared to the bulk environment, the micro-zones around the rhizosphere harbored higher abundances of ARGs in some plants, such as soybean, rice, lettuce, maize, and ryegrass [41]. All 13 ARGs, including *tetA*, *tetG*, *tetO*, *tetQ*, *tetW*, *sul1*, *sul2, blaOXA-1*, *blaPSE*, *blaTEM*, *ermA*, *ermB*, and *ermF*, detected in livestock wastewater were also found in water dropwort, verifying the close correlation of ARGs between the plants and their growth environments [14,16,42]. In this study, we found the abundance of tetracycline antibiotics was higher than that of the other antibiotics in both livestock wastewater and water dropwort. This would induce a stricter selective pressure on the emergence and enrichment of ARGs encoding resistance to tetracycline antibiotics in these environments. Therefore, compared to the other ARGs in this study, the abundance of ARGs encoding resistance to tetracycline antibiotics was higher, especially that of *tetW* with 6.85 × 10^11^, 2.81 × 10^10^, and 4.66 × 10^9^ copies g^−1^ in livestock wastewater, roots, and stems and leaves, respectively (Figure 3). In this case study, the higher abundance of ARGs encoding resistance to tetracycline antibiotics than that of the other genes might be attributed to the higher residual concentration of tetracycline antibiotics, as well as the highly sensitive nature of bacteria in wastewater to tetracycline antibiotics. Twelve ARGs were more abundant in the livestock wastewater than in the water dropwort, except *sul2*, and the abundance of all thirteen ARGs was much higher in the roots than in the stems and leaves. 

**Figure 4:**
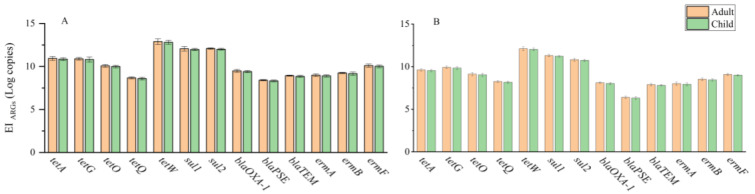
The estimated daily intake of ARGs (EI_ARGs_) for adults and children from consuming contaminated water dropwort roots (**A**) or stems/leaves (**B**).

The EI_ARGs_ is used to assess the health risks of ARGs in agricultural products [14,43]. In this study, the EI_ARGs_ of roots for adults ranged from 2.57 × 10^8^ to 7.75 × 10^12^ copies g^−1^, while the EI_ARGs_ of roots for children ranged from 2.12 × 10^8^ to 6.40 × 10^12^ copies g^−1^. The EI_ARGs_ of stems and leaves for adults ranged from 2.50 × 10^6^ to 1.29 × 10^12^ copies g^−1^, while the EI_ARGs_ of stems and leaves for children ranged from 2.06 × 10^6^ to 1.06 × 10^12^ copies g^−1^. In general, the EI_ARGs_ values of roots were higher than those of stems and leaves (Figure 4). Although the exact effects and mechanisms of ingesting exogenous ARGs on human health are unclear, many studies have indicated that intestinal microbiota can acquire exogenous ARGs via horizontal gene transfer [14,15,16,17]. If exogenous ARGs are acquired by pathogenic bacteria, it would be detrimental to curing diseases. Therefore, our results suggested that the ARGs’ contamination of the water dropwort used to purify livestock wastewater should be paid more attention.

## 4. Conclusions

A case study was conducted to explore how antibiotics bioaccumulate and the abundance of ARGs change in water dropwort grown in livestock wastewater. Furthermore, their potential risks posed to human health were also assessed. Water dropwort absorbed and bioaccumulated considerable amounts of antibiotics from livestock wastewater. The concentrations of antibiotics varied among the organs of water dropwort and were higher in the roots than in the stems and leaves. The human health risk assessment showed that the ratios of EDI to ADI of the antibiotics in water dropwort were below the safety threshold. This case study indicated that the consumption of water dropwort would be safe when considering the intake of the antibiotics. However, further case studies should be conducted to provide more data to comprehensively evaluate the health risks of the water dropwort used to purify livestock wastewater. All 13 ARGs detected in the livestock wastewater were also found in the water dropwort, suggesting the close correlation of ARGs between livestock wastewater and water dropwort. Tetracycline resistance genes were much more abundant than the other ARGs in livestock wastewater and water dropwort. The estimated daily intake of ARGs for human was calculated with a wide range from 2.06 × 10^6^ to 7.75 × 10^12^ copies g^−1^. Once exogenous ARGs are acquired by pathogenic bacteria, it would be detrimental to curing diseases. Our study provides a perspective to assess the safety of consuming water dropwort used to purify livestock wastewater considering accumulated antibiotics and antibiotic resistance genes.

## Figures and Tables

**Table 1:** Information of 14 genes’ primers.

Number	Gene Name	Forward Primer	Reverse Primer	Classification
1	*16S rRNA*	GGGTTGCGCTCGTTGC	ATGGYTGTCGTCAGCTCGTG	
2	*blaOXA1*	CGGATGGTTTGAAGGGTTTATTAT	TCTTGGCTTTTATGCTTGATGTTAA	Beta-lactamase
3	*blaPSE*	TTGTGACCTATTCCCCTGTAATAGAA	TGCGAAGCACGCATCATC	Beta-lactamase
4	*blaTEM*	AGCATCTTACGGATGGCATGA	TCCTCCGATCGTTGTCAGAAGT	Beta-lactamase
5	*ermA*	TTGAGAAGGGATTTGCGAAAAG	ATATCCATCTCCACCATTAATAGTAAACC	MLSB
6	*ermB*	TAAAGGGCATTTAACGACGAAACT	TTTATACCTCTGTTTGTTAGGGAATTGAA	MLSB
7	*ermF*	CAGCTTTGGTTGAACATTTACGAA	AAATTCCTAAAATCACAACCGACAA	MLSB
8	*sul1*	CAGCGCTATGCGCTCAAG	ATCCCGCTGCGCTGAGT	Sulfonamide
9	*sul2*	TCATCTGCCAAACTCGTCGTTA	GTCAAAGAACGCCGCAATGT	Sulfonamide
10	*tetA-01*	GCTGTTTGTTCTGCCGGAAA	GGTTAAGTTCCTTGAACGCAAACT	Tetracycline
11	*tetG-01*	TCAACCATTGCCGATTCGA	TGGCCCGGCAATCATG	Tetracycline
12	*tetO-01*	ATGTGGATACTACAACGCATGAGATT	TGCCTCCACATGATATTTTTCCT	Tetracycline
13	*tetQ*	CGCCTCAGAAGTAAGTTCATACACTAAG	TCGTTCATGCGGATATTATCAGAAT	Tetracycline
14	*tetW*	GAGAGCCTGCTATATGCCAGC	GGGCGTATCCACAATGTTAAC	Tetracycline

## Data Availability

All the data supporting this article are included in the main text.

## Supplementary Materials

The following supporting information can be downloaded at https://www.mdpi.com/article/10.3390/antibiotics11040428/s1, Table S1: The concentrations of antibiotics in livestock wastewater and water dropwort. Different letters indicate significant differences between livestock wastewater, roots, and stems and leaves (*p* < 0.05); Table S2: The antibiotic enrichment capacities of roots and stems/leaves. Different letters indicate significant differences between livestock wastewater, roots, and stems and leaves (*p* < 0.05). BAF = C_plant_/C_wastewater_, where C_plant_ is the concentration of roots or stems and leaves and C_wastewater_ is the concentration of livestock wastewater.
