# Supplementary material for: The Safety of Consuming Water Dropwort Used to Purify Livestock Wastewater Considering Accumulated Antibiotics and Antibiotic Resistance Genes
Source: Antibiotics (Basel). 2022 Mar 23;11(4):428. doi: 10.3390/antibiotics11040428 (PMC9031747; doi:10.3390/antibiotics11040428)
Supplement: Supplementary file 1 [file antibiotics-11-00428-s001.zip › antibiotics-1579884-supplementary.pdf]

antibiotics

Supplementary materials of the article:

The Safety of Consuming Water Dropwort Used to Purify Livestock Wastewater Considering Accumulated Antibiotics and Antibiotic Resistance Genes

Dongrui Yao [1,†], Yajun Chang [1,†], Wei Wang [1], Linhe Sun [1], Jixiang Liu [1], Huijun Zhao [2] and Weiguo Zhang [3,*]

[1] Jiangsu Key Laboratory for the Research and Utilization of Plant Resources, Institute of Botany, Jiangsu Province and Chinese Academy of Sciences (Nanjing Botanical Garden Mem. Sun Yat-Sen), Nanjing 210014, China; yaodongrui@cnbg.net (D.Y.); changyj@cnbg.net (Y.C.); izjwang@163.com(W.W.); linhesun@cnbg.net (L.S.); ljx891654338@163.com (J.L.)
[2] College of Geography and Environmental Science, Northwest Normal University, Lanzhou 730070, China; zhaohj0707@163.com (H.Z.)
[3] Institute of Agricultural Resources and Environment, Jiangsu Academy of Agricultural Sciences, Nanjing 210014, China
* Correspondence: weiguozhang@jaas.ac.cn; Tel: +86-25-2584390581.
† These authors contributed equally to this work.

Citation: Yao, D.; Chang, Y.; Wang, W.; Sun, L.; Liu, J.; Zhao, H.; Zhang, W. The Safety of Consuming Water Dropwort Used to Purify Livestock Wastewater Considering Accumulated Antibiotics and Antibiotic Resistance Genes. *Antibiotics* **2022**, *11*, 428. https://doi.org/10.3390/antibiotics11040428

Academic Editors: Paul I. Kadetz and Thérèse Hesketh

Received: 23 January 2022
Accepted: 19 March 2022
Published: 23 March 2022

Publisher's Note: MDPI stays neutral with regard to jurisdictional claims in published maps and institutional affiliations.

Copyright: © 2022 by the authors. Licensee MDPI, Basel, Switzerland. This article is an open access article distributed under the terms and conditions of the Creative Commons Attribution (CC BY) license (https://creativecommons.org/licenses/by/4.0/).

Table S1. The concentrations of antibiotics in livestock wastewater and water dropwort. Different letters indicate significant differences between livestock wastewater, roots, and stems/leaves ($p < 0.05$).

Antibiotic concentration (ng g^{-1})	Wastewater	Root	Stem/leaf
Sulfamethazine	0.043 ± 0.003 [a]	0.148 ± 0.012 [b]	0.132 ± 0.009 [b]
Oxytetracycline	0.238 ± 0.012 [a]	6.719 ± 0.56 [b]	3.149 ± 0.231 [c]
Doxycycline	0.341 ± 0.025 [a]	10.390 ± 0.71 [b]	2.892 ± 0.110 [c]
Tetracycline	0.326 ± 0.033 [a]	9.779 ± 0.762 [b]	2.603 ± 0.217 [c]
Chlortetracycline	0.305 ± 0.019 [a]	3.857 ± 0.193 [b]	4.254 ± 0.266 [b]
Azithromycin	0.031 ± 0.007 [a]	0.179 ± 0.009 [b]	0.182 ± 0.013 [b]
Tylosin	0.065 ± 0.013 [a]	0.898 ± 0.127 [b]	0.973 ± 0.119 [b]

Table S2. The antibiotics enrichment capacities of roots and stems/leaves. Different letters indicate significant differences between livestock wastewater, roots, and stems/leaves ($p < 0.05$). BAF = $C_{plant}/C_{wastewater}$, where C_{plant} is the concentration of roots or stems/leaves, and $C_{wastewater}$ is the concentration of livestock wastewater.

BAF	Root	Stem/leaf
Sulfamethazine	3.424 ± 0.231 [a]	3.036 ± 0.173 [a]
Oxytetracycline	28.233 ± 1.23 [a]	13.233 ± 0.557 [b]
Doxycycline	30.469 ± 2.91 [a]	8.481 ± 1.16 [b]
Tetracycline	30.023 ± 3.62 [a]	7.990 ± 1.44 [b]
Chlortetracycline	12.651 ± 1.79 [a]	13.954 ± 2.01 [a]
Azithromycin	5.795 ± 0.893 [a]	5.863 ± 0.672 [a]
Tylosin	13.818 ± 1.21 [a]	14.968±0.966 [a]
